# Implementation gaps in culturally responsive care for refugee and migrant maternal health in New South Wales, Australia

**DOI:** 10.1186/s12913-023-09066-7

**Published:** 2023-01-17

**Authors:** Katarzyna Olcoń, Delia Rambaldini-Gooding, Chris Degeling

**Affiliations:** School of Health and Society, Faculty of the Arts, Social Sciences and Humanities, The University of Wollongong, Wollongong, NSW 2522 Australia

**Keywords:** Refugee women, Migrant women, Maternal health, Service providers, Health policy, Cultural competence, Culturally responsive care, Moral distress

## Abstract

**Background:**

Refugee and migrant women are at higher risk of childbirth complications and generally poorer pregnancy outcomes. They also report lower satisfaction with pregnancy care because of language barriers, perceived negative attitudes among service providers, and a lack of understanding of refugee and migrant women’s needs. This study juxtaposes health policy expectations in New South Wales (NSW), Australia on pregnancy and maternity care and cultural responsiveness and the experiences of maternal healthcare providers in their day-to-day work with refugee and migrant women from non-English speaking backgrounds.

**Methods:**

This study used a qualitative framework method to allow for a comparison of providers’ experiences with the policy expectations. Sixteen maternal health service providers who work with refugee and migrant women were recruited from two local health districts in New South Wales, Australia and interviewed (November 2019 to August 2020) about their experiences and the challenges they faced. In addition, a systematic search was conducted for policy documents related to the provision of maternal health care to refugee and migrant women on a state and federal level and five policies were included in the analysis.

**Results:**

Framework analysis revealed structural barriers to culturally responsive service provision and the differential impacts of implementation gaps that impede appropriate care resulting in moral distress. Rather than being the programmatic outcome of well-resourced policies, the enactment of cultural responsiveness in the settings studied relied primarily on the intuitions and personal responses of individual service providers such as nurses and social workers.

**Conclusion:**

Authentic culturally responsive care requires healthcare organisations to do more than provide staff training. To better promote service user and staff satisfaction and wellbeing, organisations need to embed structures to respond to the needs of refugee and migrant communities in the maternal health sector and beyond.

**Supplementary Information:**

The online version contains supplementary material available at 10.1186/s12913-023-09066-7.

## Introduction

The latest data indicate that more than a quarter (27%) of mothers who give birth in Australia were born in a mainly non-English speaking country [1]. The provision of culturally and linguistically appropriate care is central to optimal health service provision to refugee and migrant groups, particularly to women during the perinatal period [2, 3]. The life experiences, socioeconomic position and legal status of these two groups are very different, but both can encounter problems when accessing perinatal care. Professional standards and various multicultural health policies emphasize the need for cultural competency, safety, and sensitivity in healthcare provision [4, 5]. Despite this institutional attention to the importance of culturally appropriate care, refugee and migrant women from non-English speaking backgrounds in Australia have poorer pregnancy outcomes [6, 7] and lower satisfaction with pregnancy care than women from the general population [2, 8].

The provision of culturally and linguistically appropriate care is challenging starting with the ambiguity of terms and lack of clear definitions in healthcare policy and practice [9]. The term “cultural competence” has been widely used in many countries, yet it risks implying that “culture can be reduced to a technical skill for which clinicians can be trained to develop expertise” ([10], p.1673). Failing to recognise that migrant and refugee women inhabit multiple social dimensions including race, gender, and class [11, 12], “cultural competence” reduces patients to stereotypical cultural characteristics, which in turn continues to reproduce the existing social inequality [13]. In Australia the term “cultural safety” has gained popularity in healthcare policy and practice particularly to capture the need to tailor service provision to Indigenous communities [14]. In this article, however, we will use the term “cultural responsiveness” or “culturally responsive care” [15] as it is more commonly used in relation to refugee and migrant communities [16]. Based on the concept analysis by Smith et al. [15], cultural responsiveness encompasses extrinsic and self-knowledge, inclusive relationships, cultural respect, social justice/human rights, and self-reflection including the awareness of power and privilege. Noting the limitations of the culture-oriented discourse in healthcare [17], we believe “cultural responsiveness” is better able to challenges the linear model such as cultural competence and is cognizant of power imbalances, both between the practitioner and the client and in the broader society.

When it comes to implementation, studies in Australia, Canada, and the United States show that cultural responsiveness extends beyond the responsibility and capability of an individual provider. It requires a shift in organisational culture to one that is authentically committed to culturally responsive care [18–20]. However, in the Australian health policy context women from refugee and migrant backgrounds are often treated in the same category. Furthermore, the way in which providers interpret relevant healthcare policies and implement services determines how refugee and migrant women from non-English speaking backgrounds experience maternal health care [21]. The potential institutional and structural barriers to cultural responsiveness and how those impact maternal service providers, therefore, require further attention. Specifically, social and healthcare service providers can experience moral distress – circumstances where care providers “know the right thing to do, but institutional constraints make it nearly impossible to pursue the right course of action” ([22], p. 6). Moral distress extends our understanding of the drivers and impacts of ‘burnout’ among care providers by drawing attention to tensions between their requisite responsibility to uphold professional standards and the institutional barriers that preclude them from doing so.

The experience of moral distress in social and health service providers including nurses and social workers is well documented [23–28]. Although some new studies have emerged about the experience of moral distress in midwifes in Ghana and Iran [29, 30], no studies focus on the challenges experienced by maternal health service providers in delivering culturally responsive care and the subsequent experiences of moral distress. Drawing on the policy conceptualisation of culturally responsive care as well as the literature on moral distress, this article explores the link between local and national healthcare policies that seek to ensure the provision of culturally responsive care and experiences of moral distress in maternal health service providers in New South Wales (NSW), Australia.

## Study background

### Vulnerability of pregnant refugee and migrant women

Refugee and migrant women experience a range of health risk factors including adverse socioeconomic position, social isolation, racism, and discrimination, and in many cases posttraumatic stress disorder [31–33]. Pregnant women from refugee and migrant backgrounds are considered a particularly vulnerable group, often suffering from complex medical and psychosocial problems, and facing cultural and language barriers in accessing antenatal care [7, 18, 34]. Mothers born in non-English speaking countries are also found to commence antenatal care in NSW later than mothers born in English-speaking countries [35]. As a result, refugee and migrant women in Australia are at higher risk of childbirth complications and generally poorer pregnancy outcomes including preterm birth, stillbirth, congenital anomalies, and maternal mortality, consistent with evidence from other parts of the world [6, 36]. Systematic reviews and meta-analyses indicate the major negative factors affecting provider–client interactions in maternal health services were language barriers and healthcare professionals’ lack of cultural sensitivity resulting in poor client-provider relationships, ineffective communication, discrimination, cultural clashes, and negative experiences of clinical intervention [19, 37–39]. Refugee and migrant women often have different perceptions related to sexual and reproductive health associated with family planning and pregnancy. This requires healthcare professionals to understand cultural relativism, the social determinants of health, and how lived experiences influence the conceptualization of family planning [40].

Although many culturally and linguistically diverse women find Australian maternity and sexual health service providers welcoming, kind and “providing care equally to all patients regardless of their social status” ([31], p. 305), several other Australian studies have found the opposite trends [6, 41]. In particular, providers often lack adequate cultural knowledge, some hold stereotypical assumptions, and the healthcare system in general is not culturally responsive. Women’s dissatisfaction with pregnancy care results from a perceived lack of understanding amongst providers of the women’s needs, language barriers, inability to attend regular appointments, poor social connections, and perceived negative attitudes of service providers [3, 42, 43]. The experiences related to labour and postnatal period caused challenges for refugee and migrant women, including failing to take into consideration issues that were important to them or keep them informed about what was happening during the birth [8]. Other identified issues include the lack of continuity of the specialized multicultural care women might have received during pregnancy [32], a lack of interpreters, and the unwanted presence of male doctors and students [2, 7].

### Refugee and migrant maternal health policy context

Maternal health services are provided free to women who are refugees, Australian citizens, and residents – however, temporary visa holders are liable for any medical costs incurred in Australia. The Australian Department of Home Affairs recommends all temporary visa holders take out private health insurance, and private health insurance is a prerequisite for some temporary visa types. Most of private insurance companies have a 12-month pregnancy waiting period and even once the benefits are available, the cost of pregnancy and birth care varies significantly. Australian healthcare has a decentralised organisational and funding structure, such that both federal and provincial policies operate to shape care [44]. Because migrant and refugee women receive maternal and perinatal care in mainstream health services there are two sets of relevant policies: those that pertain to all women [45, 46] and those that are specific to the provision of care through specialised services to women from refugee and migrant backgrounds [4, 16, 47].

We note that except for the NSW Refugee Health Plan, which focuses on refugee women specifically, policy directives and care guidelines treat refugee and migrant women within the same category, e.g., Pregnancy Care for Migrant and Refugee Women [4] or situate them within the term “culturally and linguistically diverse (CALD)”, e.g., NSW Plan for Healthy Culturally and Linguistically Diverse Communities [16]. These key recommendations are predicated on the acknowledgement that migrant and refugee women’s experiences are diverse, but many may face issues related to resettlement that impact on the uptake of antenatal care and pregnancy outcomes. We will thus use the term “refugee and migrant women” in this paper, understanding its potential limitations.

Against this background an overarching goal of maternal health services in Australia is to provide a woman-centred approach that aims to ensure that a woman’s needs and expectations, including cultural ones, are considered and respected under the universal health system [45]. Nevertheless, we know little about the enactment of these policies in practice and the experiences of the providers who are expected to provide culturally responsive care to refugee and migrant women. The experiences and challenges of providers who work with marginalized groups, such as refugee and migrant women, require special examination given the constraints and pressures they experience including the need to deliver linguistically and culturally appropriate services. These greater demands are likely to increase risk of burnout and moral distress [48] and subsequently lower the quality of care. This study aimed to capture the day-to-day experiences of maternal health service providers including nurses, midwifes, and social workers in their work with refugee and migrant women from non-English speaking backgrounds within the context of how they seek to operationalize policies aimed at enacting culturally responsive care. In doing so, we sought to understand to what extent the policies are reflected in practice and the potential enablers and barriers to implementing these policies. We were further interested in exploring whether there are any links between the expectations of culturally responsive care, organisational capacity and commitment to cultural responsiveness, and providers’ experiences of moral distress.

## Methods

### Review of relevant national and local policies

A systematic search was conducted for policy documents related to the provision of maternal health care to refugee and migrant women on a state and federal level. Specifically, we searched the NSW Ministry of Health and the Australian Government Department of Health websites to identify relevant documents. Five policies were included in the analysis: two policies that pertain to all women and three that are specific to the provision of care through specialised services to women from refugee and migrant backgrounds (see Table 1 in Supplementary Materials for details).

### Study setting and interview participant recruitment

The maternal health service staff who took part in this study work in two local health districts—one in outer metropolitan Sydney and one a regional area with a substantial urban population but also smaller towns and settlements. The migrant population of the outer metropolitan area is approximately 45% with most of the language other than English speakers from Iraq, India, Vietnam, Lebanon, Fiji, and China. The migrant population of the regional area is approximately 25%, with the majority of language other than English speakers from the Former Yugoslav Republic of Macedonia and China [49]. The refugee population differs significantly to the migrant population in both areas. Before the COVID-19 pandemic severely restricted Australia’s migrant and refugee intake in 2020, approximately, 2300 refugees settled in the outer metropolitan area per year mainly from Iraq, Iran, Afghanistan, Syria, and Burma [50]. Approximately, 286 refugees settled in the regional area per year mainly from the Congo, Syria, Iraq, Eritrea, Burma, Ethiopia, Burundi, and Iran [50].

The outer metropolitan local health district has five hospitals, four of which provide the full range of maternity health services and post-natal care (up to 6 weeks). Ante-natal care is provided through a combination of hospital-based care, general practitioner (GP) shared care or midwifery group practice depending on women’s health status. The regional local health district has four hospitals, the largest of which offers antenatal services, an early assessment unit, birthing unit, midwifery-led care unit and post-natal care (up to 6 weeks). The second largest of the hospitals only provides maternity services for low-risk pregnancies and post-natal care. The two other hospitals only offer antenatal services through GP shared care and midwife outreach clinics located in the community. The maternity social work team also operates at hospitals with birthing units. In both local health districts, refugee and migrant specific health services operate independently of mainstream maternity services and there are no formal referral pathways.

Given the inter-professional nature of maternal healthcare, we sought the perspectives of a range of healthcare providers. To identify potential interview participants, a list of mainstream and refugee/migrant specific agencies, clinics, institutions, and organisations that operate in the regional and metropolitan local health districts in NSW that comprise the study setting was compiled. We contacted the site directors to discuss the project and after receiving permission, we recruited potential participants from those sites through flyers and electronic list-serves. In the case of the latter, we sent recruitment emails to list-serve owners, who distributed the email following approval. All participants provided written consent for participation, and the Human Research Ethics Committee of the University of Wollongong and the local health districts of interest granted ethics approvals.

### Participant characteristics

In-depth interviews were conducted with 16 providers who work with refugee/migrant women in the context of maternal health services: seven nurses, two midwifes, five social workers, and two multicultural health workers. Nine of the providers worked in refugee specific/multicultural health organisations and seven worked in mainstream services. Fifteen of them were females and one was a male. They identified with the following cultural backgrounds six were White Australian, four were Middle Eastern, three were White European, two were Asian, and one was African. The composition of the sample is reflective of the health workforce in Australia [51]. To protect the confidentiality of participants, we do not identify their cultural identity when reporting the results and describe their professional roles in broad terms. Interviews took place from November 2019 to August 2020. All participants took part in semi-structured interviews that explored their experiences working with refugee/migrant women, and their perceptions of the challenges faced. Interviews focused on understanding provider perspectives on the barriers to culturally responsive services and drawing out detailed and specific accounts of the broader impacts of these policies and institutional constraints on provider experiences of care provision. The interviews were conducted by the first and the second author in a location that was comfortable for the participant, usually their work office. Due to COVID-19 restrictions, the final interview was conducted via Zoom. The interviews were audiotaped and later transcribed. They ranged from 31 to 70 min with an average length of 60 min. Detailed fieldnotes were recorded following each interview.

### Data analysis

All data (interview transcripts, policy documents, and fieldnotes) were analyzed thematically by the first two authors [52]. The first and the second author read the transcripts and materials several times, identifying minor and major codes and the relationships between them using QSR NVivo 12 software. Following the precepts of framework methodologies, all of this information was entered into a separate tabular matrix of rows (cases), columns (codes) and ‘cells’ of summarised data [53]. The matrices provide a structure into which the researcher can systematically summarise coded data for inductive synthesis and aide deductive contrast and comparison [52, 53]. Preliminary analysis suggested that moral distress in providers is an outcome of structural constraints and driven by policy dimensions. In addition to the initial set of inductive codes, our analytical framework included deductive categories based on the recommendations contained in the suite of guiding federal and NSW policies. They are: 1) understanding the needs, experiences and identities of refugee and migrant women, 2) improving access and quality of services, 3) taking an individualised approach, and 4) using interpreters. We systematically mapped the whole dataset against these categories to determine if providers perceive themselves to be adequately supported and sufficiently resourced to follow these policies and guidelines.

To ensure rigor in our analysis, we held regular debrief meetings to discuss any potential biases and enhance the conceptual development of the study [54]. We also engaged in member checking, which included inviting participants to check their interview transcriptions, facilitating member checking sessions to receive feedback on the study findings and presenting the results to all stakeholders, followed by an opportunity for additional feedback [55]. The final stage of analysis drew on the knowledge and expertise of the team to test alternative hypotheses and refine our insights through discussion between authorship team and in the process of revising drafts.

## Results

The policy review confirmed that an explicit goal shared by all policies relevant to all maternal health services is to promote a woman-centred approach to take into consideration individual social, cultural, physical, psychological and spiritual needs, to improve the experience of antenatal, birth, and perinatal care. This goal is operationalised in both general maternal health service and refugee and migrant specific maternal health service policies as a set of recommendations to simultaneously: 1) ensure that refugee and migrant women and their families are provided with enough information in a culturally responsive manner to make informed choices about pregnancy care; and 2) respect women’s individual needs and care choices.

The Commonwealth and NSW guidelines for general maternity services [45, 46] provide recommendations to help to address the needs of service users through:

- recognising the importance of multicultural health workers to assist women to navigate the healthcare system,
- the provision of healthcare interpreters,
- the adoption of an individualised approach informed by cultural awareness and understanding, and
- the provision of culturally appropriate information and resources to enable women to make informed choices about their care.

As to local policies, the NSW Plan for Healthy Culturally and Linguistically Diverse (CALD) Communities [16] aims to ensure that mainstream services provide culturally responsive care to CALD communities. The four outcomes are to improve access to and quality of care for CALD communities, build health literacy among CALD communities to ensure they can make informed choices about their healthcare, be responsive to individual’s needs, language, and culture, and understand the experiences and identities of CALD communities.

Turning to refugee and migrant specific guidelines, the current NSW Refugee Health Plan [47] focuses on delivering quality care through refugee-specific services and “referral to culturally competent mainstream health services” (p. 24). Eight strategic priorities are detailed including developing health plans and policies that prioritise refugees; working in collaboration with -general practitioners and other health professionals to ensure newly arrived refugees and humanitarian migrants have access to health assessments and follow-up; providing specialised refugee health services; and providing high quality care to refugees within mainstream services [47].

Against this background, during analysis of the interviews it became clear that providers faced a variety of challenges when working cross-culturally. Their efforts to implement relevant cross-cultural policies such as improving access and quality of services, taking an individualised approach, and using interpreters were hindered by structural and institutional constraints. In what follows, the findings are organized to clearly describe how providers work within and respond in their day-to-day practices to each set of policy recommendations (see Table 2 in Supplementary Materials for details). We then highlight the major similarities and differences in the experiences of the providers working in mainstream services and those affiliated with refugee/migrant specific organisations. We also include some of the providers’ recommendations offered during the interviews.

### Understanding the needs, experiences and identities of refugee and migrant women

Policies and guidelines relevant to maternal health services in Australia emphasize that women should receive care that is clinically safe and culturally responsive. To achieve this, service providers in NSW are required to understand and accommodate, where possible, the needs, experiences, and identities of refugee and migrant women from non-English speaking backgrounds [16]. In concrete terms, improving the experiences of antenatal care among migrant and refugee women can involve the introduction of new roles, activities, and measures such as: appointing ethnic-specific cultural liaison officers, establishing women’s groups to maintain cultural connections, developing knowledge of cultural traditions and practices relevant to pregnancy and birth, and adopting “a cross-cultural approach to communication” ([4], p7). During interviews, providers told us that efforts to meet these expectations revolved around attempts to create a safe and comfortable environment, being curious about the patient’s culture, being “extra kind” and compassionate, having “extra warmth”, and remembering that the women are the experts on their lives. Most importantly, when working with clients from refugee and migrant backgrounds, providers needed to avoid making assumptions and creating a perception that they were making negative judgement of other cultures.

Meeting the requirements for culturally responsive care, however, was challenging. Service providers described how the goals of appropriate clinical care and cultural respect were frequently brought into tension, resulting in both practical and moral dilemmas for staff and their managers. For example, several providers described how they struggled to accommodate and safely manage cultural practices important to their clients such as co-sleeping or use of jewellery (e.g., bracelets or crosses), that they believed might increase the risk of infant death. The NSW Health Maternity Care Policy [46] explicitly outlines safe sleeping recommendations that advise that infants sleep in their own cot or bassinet. Some providers reluctantly accepted co-sleeping with a child as culturally appropriate whereas others recounted instances of having advised parents that their decision to maintain this practice may require them to make a child protection report. Service providers also spoke of the difficulties in accepting and accommodating the presence and the role of male partners, often viewed as unsupportive and dominating, in service encounters. Again, the responses varied from viewing the partner’s presence as culturally appropriate to asking the male to leave the room and only talking to the woman. Difficulties adjusting care to refugee and migrant women extended to mandated protocols and assessment instruments. Although the Australian Government’s Pregnancy Care Guidelines [45] acknowledge the inappropriateness of several psychosocial maternal assessment tools for use with women from migrant and refugee backgrounds, many remain in use. For example, some providers questioned the cultural relevance and appropriateness of the widely used Edinburgh Postnatal Depression Scale (EPDS), which includes items that are not easily translatable to another language and/or cultural understanding.

Providers also described witnessing the displays of judgement and bias in their co-workers in mainstream settings – particularly when working in multidisciplinary teams. Others sought to take the onus of responsibility for providing culturally responsive care off individual providers, noting that the entire healthcare system could be at times patronizing, racist, and damaging to refugee and migrant women. As one participant reported: “There's a lot of racism in health sectors. I've seen it quite often working as a midwife.” Even though the Australian healthcare organisations acknowledge a responsibility to prepare and support their staff to deliver culturally responsive services [4, 16], the providers we spoke with, particularly those who worked in mainstream services, indicated that the “cultural competence training” available to them was inadequate and not improving standards of care. The current mandatory online cultural competence packages were described as “perfunctory”, and “a one-off thing” that, in the opinion of participants, risked harmfully categorizing groups of people. Providers worried about the dangers of making assumptions based on someone’s culture and the arrogance embedded in the term “cultural competence.” The training also did not address issues of racism.

For these reasons many providers across settings believed that more needed to be done to increase the cultural responsiveness of the services offered to the refugee and migrant women at an organisational level, especially in hospital settings. There were, however, different perspectives on what “cultural competence” training should entail and whether it should be mandatory. Those against mandatory trainings argued that these programs are ‘tick-and-flick’ requirements that do not change anything; what is required is a genuine interest in someone else’s culture, traditions, and lived experience. Whereas those in favour argued that this type of training is at least a start at getting where services needed to be.

### Improving access and quality of services

For participants who worked at refugee/migrant specific organisations providing access to quality healthcare for refugee and migrant clients was a major challenge. They described their clients’ cases as being highly complex and requiring an active and holistic management including having to “fight” for their clients’ access to quality services. Part of the problem was that their case records were not linked to those held by mainstream health services. They felt isolated in their role at times, because, as one refugee health nurse noted: “No one ever knows who we are or what we’re doing.” Mainstream providers, on the other hand, reported that the specialised refugee/migrant health services were not utilized properly because many of their colleagues lacked awareness of their existence or role. They also did not understand the rights of asylum seekers thus often denying their access to care.

Providers explained that although hospitals made some attempts at providing culturally responsive services (e.g., accommodating families who need to bury a dead child on the same day, allowing visits of religious leaders), there were a variety of structural factors that limited their ability to respond to cultural needs such as staff or space limitations (e.g., lack of female doctors or inability to host large families in small hospital rooms). Hospitals also usually lacked bilingual/multicultural obstetric liaison officers and birthing classes. The policy directives of providing access and quality of services to refugee and migrant women were thus constrained by inadequate resource allocation and fragmented communication and referral pathways. As one midwife summarized: “From a service perspective, I think we can do so much better. I think we can do so much better for those women.” Participant recommendations on how to ease some of the challenges they experienced in working with refugee and migrant women included hiring more staff including female doctors, better referral pathways and coordination of services to overcome the disconnect between the refugee/migrant specific organisations, and better supervision and support when dealing with complex cases.

The mainstream providers also emphasised they were aware of the range of external barriers refugee and migrant women faced when coming to a hospital, including lack of transport and childcare. The women were also frequently dealing with poverty, health issues, and social isolation, which as one nurse explained made their lives “just really, really precarious”. She went on to describe how these challenges, although beyond providers’ control, would often create a sense of burden and powerlessness in the staff: “When you have that constantly with family after family, it can be really hard to maintain your own sense of well-being and optimism about the future.”

### Taking an individualised approach

Taking an individualised approach to the provision of maternal healthcare was portrayed in relevant policies as integral to ensuring woman-centred and culturally responsive care [4, 45]. Both mainstream and refugee/migrant specific providers acknowledged that taking the time to build trust and establish a relationship with a woman is essential. They believed it is important to be aware of cultural practices related to the perinatal period and maternity care provided in a woman’s home country and take the time to learn about a woman’s family background and level of support available. Attaining this goal was viewed as more challenging in mainstream settings due to the shorter timeframe a woman is engaged with services, limited appointment times, and persistent staff shortages that impacted on the time service providers can spend with each client. Mainstream service providers also noted that some organisational regulations and legislation such as mandatory reporting requirements can conflict with cultural practices (e.g., co-sleeping) thus making it difficult to build rapport and trust with a woman.

The individualised approach in maternal health services was also crucial to respond to the complex needs of many refugee and migrant women related to experiences of trauma, including sexual violence. Some providers reported that support services offered by the NSW government to assist new mothers with their mental health are not adequately resourced and equipped to assist refugee and migrant women with complex trauma. Providers believed that successfully meeting the needs of these women would require better coordination and communication between mainstream and refugee/migrant specific services and most importantly adopting a trauma-informed approach across health settings and policies.

Finally, the NSW Ministry of Health [16] policy indicates that provision of individualised care involves women being actively involved in decisions about their health. However, some providers highlighted that women from refugee and migrant backgrounds often have less opportunity for education resulting in low health literacy and subsequently relying on their male partners to articulate their health concerns. Providers’ recommendation was to hire bilingual/multicultural obstetric liaison officers to help women express their voice. Some providers also noted that women’s resilience and resourcefulness are not adequately acknowledged in the health policies and underestimated in practice. A social worker described this deficit perspective and “saving” attitude in the following way:

It’s important for us to remind ourselves that we are not here to save them. They don’t need saving. We’re here to support them and be that guiding hand when they need it. Because they have survived all this time without us. They have the tools themselves.

Although providers saw the value of taking the time to listen and learn from the women’s experiences and “not assume we know best”, their heavy workload would often prevent following these guidelines.

### Using interpreters

The use of healthcare interpreters featured in all refugee and migrant specific health policies. All healthcare providers have around-the-clock access to interpreters through the telephone-based *Commonwealth Translating and Interpreting Service (TIS)*. Integrating this service into the day-to-day work of providers was often challenging, especially if clients were experiencing distress such as during labour or pregnancy complications. Providers identified specific issues with TIS such as poor telephone connection and interpreters being preoccupied (e.g., collecting children from school). They also reported that doctors may rush the call in emergency situations or misunderstand clients who were unable to articulate their health concerns to interpreters (e.g. where they feel pain and point to a body part). Face-to-face interpreting services were preferred by all service providers but were only available through NSW Health Interpreters during business hours. Even though relevant NSW policies mandate access to healthcare interpreters, the availability of such services differed between service providers and sites. Specifically, providers from the regional area reported rarely having access to onsite interpreters and a difficulty locating interpreters for some languages.

Individual service providers are responsible for organising healthcare interpreters for their clients [45]. Providers generally took the initiative to schedule interpreters and did not report widespread reluctance among women to accept interpreting assistance. Nevertheless, mainstream providers reported that the joint scheduling of interpreters and antenatal appointments was difficult in practice. For example, if women were late for an appointment and the interpreter could not stay or had already left, women would prefer to conduct the appointment without an interpreter rather than reschedule. In these instances, women would sometimes express a preference for using *Google Translate* or a friend/family member who has accompanied them.

All providers are directed by the Pregnancy Care for Migrant and Refugee Women Guidelines to avoid using a “woman’s partner, friends or relative to act as interpreters unless absolutely necessary” ([45], p. 6). Some participants expressed concern that informal interpreters may not understand medical terms and may translate them incorrectly. Some refugee and migrant specific providers also felt that informal interpreters may only translate what they think a woman needs to know or may make decisions on a woman’s behalf. Providers from mainstream services attempted to use healthcare interpreters as directed by the policy. However, this was not always possible, for example, if a woman had not requested an interpreter and insisted that the appointment continue without a formal interpreter. Informal interpreters were also used by when discussing matters that were not critical to the woman or baby’s care, for example, changing nappies.

In addition, in line with the Pregnancy Care for Migrant and Refugee Women [4], the use of female interpreters was preferred by service providers and by clients. Service providers reported that female interpreters may provide support to women, particularly if women do not have any family present, have disclosed a history of sexual violence, or in cases where it is culturally inappropriate for a male to be present such as during an examination or labour. However, providers reported that scheduling female interpreters was not always possible due to lack of availability.

## Discussion

Our study has revealed the policy tensions and implementation gaps maternal health service providers experience while attempting to provide culturally responsive care to refugee and migrant women. Culturally responsive care is conceptualized as a requiring a systems approach in the existing multicultural health policies in Australia [16] as well as scholarly literature [56–59]. However, the enactment of cultural responsiveness relied primarily on individual service providers such as nurses and social workers. The providers interviewed for this study made efforts to practice what they believed to be culturally responsive behaviour, by exhibiting additional kindness, warmth, and curiosity towards their refugee and migrant patients. But from their perspective, their capacity to provide optimal care to refugee and migrant women was hampered by a variety of factors beyond their control. At the point of care these included constraints around interpreting, insufficient training and supervision, and the ethnocentric norms and values embedded in many of the organisational regulations, procedures, and assessment tools. Additional institutional and structural barriers such as lack of resources, large caseloads, administrative burden, and the overall climate of economic austerity in the public sector [60–62] further limited the practitioners’ capacity to provide quality care.

Thus, maternal health service providers are expected to perform within the cultural responsiveness/competence/safety driven policy environment, yet their experience is that of being inadequately supported and insufficiently resourced to deliver culturally appropriate care. Although providers in our study operated in “doing your best for a client to the best of your ability” mode, they understood that they were providing suboptimal care to their patients. Evidence from studies in Australia with migrant and refugee women that point to high levels of dissatisfaction with maternal health services suggest that they are right [2, 8]. The cumulative result for providers of not achieving standards of culturally responsive care can be moral distress. Consistent with recent findings [63, 64], moral distress arises from problems within the organization such as an inadequate allocation of resources and lack of administrative action that inhibit providers’ capacity to practice in a culturally responsive manner. The emotional ramifications of experiencing those barriers extend beyond feeling disempowered and frustrated and can lead to exhaustion and burnout [28, 29]. On the organisational level, moral distress results in high staff turnover and retention issues [65, 66].

The comparison of the policy recommendations and the practice experience of the providers interviewed in this study, reveals that policies, focused on the provision of culturally responsive care, are often asking for “the impossible”. This results in an implementation gap between the way policy was envisioned and the way it is practiced [67]. However, as Dreachslin and Myers ([56], p. 224) state: “The bottom line is that clinicians and caregivers cannot on their own drive and follow practices that lead to culturally and linguistically appropriate care.” Without a ‘systems approach’ that considers the patients, providers, organisational policies and practices, culturally responsive care will remain an unachievable vision and a mere ‘on paper’ aspiration. To alleviate providers’ experiences of inadequacy and the resulting moral distress, a better translation of cultural responsiveness policies to practice is required. Healthcare institutions need to demonstrate an authentic commitment to culturally responsive services and an accountability in implementing systems-level responses and resolutions to ensure optimal care to refuge and migrant women.

Recent research [68, 69] goes further arguing that the structural violence and racism within the healthcare systems must be recognised as an issue that undermines equitable access to healthcare. Normalised through everyday practices, this “invisible, indirect, and insidious process” ([68], p. 1663) is inherent in European social structures, which Australia is built on. Moreover, refugee and migrant women are situated at the intersection of various marginalised identities related to race, gender, class, and often traumatic experiences [11] and are viewed in healthcare settings as “racialised and gendered bodies” ([64], p. 3). Yet structural racism and intersectionality are often not recognized within healthcare structures, policies, or professional development modules [11, 12, 14, 69]. This failure results in the current culture-oriented discourses and policies in the healthcare system and ultimately a tokenistic promotion and enactment of culturally responsive/competent care [69, 70]. If maternal health services for refugee and migrant women in Australia and other Western countries continue to be “structured around cultural difference rather than structural racism” ([70], p. 16), healthcare organisations risk continuing to produce incomplete and tokenistic policies resulting in tokenistic and suboptimal healthcare practice. Detrimental to the patients, this approach also hurts the healthcare providers. Maternal health service organisations and policymakers thus need to rethink how they conceptualize culturally responsive care, and what the enactment of cultural responsiveness looks like in practice.

Some of the additional recommendations coming from the maternal health providers included replacing or adapting the screening tools, such as the Edinburgh Postnatal Depression Scale, to make them more culturally appropriate. Informed by Western biomedical perspective, these tools may have little cultural relevance for refuge and migrant women and their universal application indicates the ethnocentric assumptions in the Australian health care system [71]. A critical review and adaptation of these and other assessment and intervention tools is beyond the reach of an individual provider and require a whole system approach. Similarly, a need for an organisational shift towards a trauma-informed approach to practice with migrant and refugee women was highlighted. In addition to the importance of introducing routine post-traumatic stress disorder screening [33], providers would benefit from training and other professional growth opportunities that focus on issues specific to refugee populations such as impact of war, displacement, experiences in refugee camps, the United Nations resettlement process, and the differences between refugee, asylum seeker and immigrant visa statuses. This is especially important given that refugee and migrant women are treated as a homogenous group, by most of the policy documents and providers. Moreover, an easier access to professional interpreters and staff training on the effective use of interpreters is required [72] to overcome the reliance on informal interpreters or online translation.

Refugee and migrant women also require greater levels of assistance with navigating the healthcare system. For example, providing orientation to prenatal services and organizing transport and baby-sitting services can enable the women to attend their appointments more easily. Finally, providers pointed to an urgent need for a better coordination and communication between mainstream services and organisations and providers that specialise in refugee and migrant care. The development of clear referral pathways between mainstream and refugee/migrant specific providers is thus required to ensure that refugee and migrant women, particularly those with complex needs, do not “fall through the cracks”.

### Limitations and implications for future research

Given the exploratory nature of the study, the purposive sampling methods, and focus on service providers from two regions only, we should avoid generalizing from these findings. It is also likely that the experiences of other professionals working with refugee and migrant women, such as general practitioners or obstetricians whom we were not able to recruit to participate in the study, or even within the same professions may vary. It is also important to note that the recruitment of maternal service providers proved difficult in the later stages of the project, which resulted in a smaller than expected sample size. This might be a result of the increased workloads of healthcare staff around the world during the COVID-19 pandemic and thus a limited capacity to participate in research [73]. Finally, the study was limited to service providers and healthcare policies only. A follow-up study is currently being conducted by the same researchers with refuge and migrant women to explore their perspectives and recommendations for maternal health services.

Given these limitations and the gaps in research on the links between cultural responsiveness policy expectations, organizational constraints, and providers’ moral distress in maternal health services, there is an ample room for future studies including a close examination of the processes driving the implementation gaps between cultural responsiveness policies and practice. Future research should also examine the role of intersectionality and structural racism in the provision on maternal health services from the perspective of both the service providers and the refugee and migrant women. A critical discourse analysis of relevant policies can provide further insight into the assumptions and values that may be reinforcing health inequities among migrant and refugee women. Finally, a co-design of an enhanced culturally responsive care strategies within healthcare setting [74] is an important next step to understand what an optimal maternal health services to refugee and migrant women might look like.

## Conclusion

By juxtaposing health policy expectations on cultural responsiveness and the experiences of maternal health care providers in their day-to-day work with refugee and migrant women, this study demonstrates the various implementation gaps in culturally responsive care. Although providers try their best to meet the needs of their refugee and migrant clients, they are often inadequately supported and insufficiently resourced to deliver culturally responsive care in the context of maternal health services. The structural and institutional constraints in implementing culturally responsive care not only result in suboptimal patient care but also drive the experience of moral distress in service providers. Authentic culturally responsive care thus requires the shift of gaze from individual providers to the healthcare organisations that can either demonstrate commitment and create supportive structures or engage in provisional and perfunctory responses to the needs of refugee and migrant communities.

## Supplementary Information

**Additional file 1: Supplementary Table 1**. Reviewed policies.

**Additional file 2: Supplementary Table 2.** Comparison of policy expectations with service providers’ experiences.

## Data Availability

The datasets used and/or analysed during the current study are available from the corresponding author on reasonable request. The data are not publicly available due to containing potentially identifying information as the study was conducted in two local health districts, one of which is located in a regional area.

## Abbreviations

NSW: New South Wales
CALD: Culturally and linguistically diverse
GP: General Practitioner
