# Supplementary material for: Implementation gaps in culturally responsive care for refugee and migrant maternal health in New South Wales, Australia
Source: BMC Health Serv Res. 2023 Jan 17;23:42. doi: 10.1186/s12913-023-09066-7 (PMC9843667; doi:10.1186/s12913-023-09066-7)
Supplement: Supplementary file 1 — Additional file 1: Supplementary Table 1. Reviewed policies. [file 12913_2023_9066_MOESM1_ESM.docx]

**Supplementary Table 1: Reviewed Policies**

| **Mainstream Policies** | | | |
| --- | --- | --- | --- |
| **Title** | **Jurisdiction and Year** | **Setting** | **Overview** |
| [Pregnancy Care Guidelines \| Australian Government Department of Health](https://www.health.gov.au/resources/pregnancy-care-guidelines) | Australian Government Department of Health (2020) | Australia-wide hospitals, GP shared care practices, midwife-led clinics providing antenatal and postnatal care | “…highlights specific approaches to pregnancy care for a range of groups, with a focus on improving the experience of antenatal care for Aboriginal and Torres Strait Islander women, migrant and refugee women and women with severe mental illness”. |
| [NSW Health Maternity Care Policy - Maternal and newborn](https://www.health.nsw.gov.au/kidsfamilies/MCFhealth/maternity/Pages/maternity-policy-review.aspx) | New South Wales Ministry of Health (2020) | NSW-wide hospitals providing GP shared care practices, midwife-led clinics providing antenatal and postnatal care | “…focuses on providing women with family-centred, quality care during pregnancy, birth and the postnatal period”. Policy is still in consultation phase. |
| **Migrant and/or Refugee Specific Policies** | | | |
| **Title** | **Jurisdiction and Year** | **Setting** | **Overview** |
| [Pregnancy care for migrant and refugee women \| Australian Government Department of Health](https://www.health.gov.au/resources/pregnancy-care-guidelines/part-a-optimising-pregnancy-care/pregnancy-care-for-migrant-and-refugee-women) | Australian Government Department of Health (2020) | Australia-wide hospitals, GP shared care practices, midwife-led clinics providing antenatal and postnatal care to migrant and refugee women | “…outlines principles of perinatal care and gives guidance on providing woman-centred and culturally safe care.. including for migrant and refugee women”. |
| [NSW Plan for Healthy Culturally and Linguistically Diverse Communities: 2019-2023](https://www1.health.nsw.gov.au/pds/Pages/doc.aspx?dn=PD2019_018) | New South Wales Ministry of Health (2019) | NSW-wide all public hospitals, public health units, local health districts, community health centres, allied health organisations, NSW Ambulance | “...strategic statewide policy for meeting the health needs of culturally and linguistically diverse consumers for the next five years. The Plan aims to ensure people of culturally and linguistically diverse backgrounds have equitable access to the health care services that are culturally responsive, safe and high quality”. |
| [Refugee Health Plan 2011-2016 (nsw.gov.au)](https://www1.health.nsw.gov.au/pds/ActivePDSDocuments/PD2011_014.pdf) | New South Wales Ministry of Health (2011) | NSW-wide specifically NSW Refugee Health Service, Service for the e Treatment and Rehabilitation of Torture and Trauma Survivors (STARTTS), Transcultural Mental Health Centre (NSW), The NSW Multicultural Health Communication Service, The Multicultural HIV/AIDS and Hep C Service, the Education Centre Against violence and the Drug and Alcohol Multicultural Education Centre, Local Health network Refugee Health Services and all public health services providing care to refugees and people with refugee-like experiences. | “…statewide plan for improving the health and well-being of refugees and people with refugee-like experiences who have settled in New South Wales. This Plan seeks to ensure the delivery of safe, high-quality services to refugees through both refugee-specific health services and through accessible, culturally and linguistically competent mainstream health services”. |
