# Supplementary material for: Implementation gaps in culturally responsive care for refugee and migrant maternal health in New South Wales, Australia
Source: BMC Health Serv Res. 2023 Jan 17;23:42. doi: 10.1186/s12913-023-09066-7 (PMC9843667; doi:10.1186/s12913-023-09066-7)
Supplement: Supplementary file 2 — Additional file 2: Supplementary Table 2. Comparison of policy expectations with service providers’ experiences. [file 12913_2023_9066_MOESM2_ESM.docx]

**Supplementary Table 2: Comparison of Policy Expectations with Service Providers’ Experiences**

| **Understanding the needs, experiences and identities of refugee and migrant women (cultural sensitivity, responsiveness, safety, competence)** | |
| --- | --- |
| **Policies say this:** | **Providers experience this (selected examples):** |
| **Cultural Safety and Sensitivity of Providers**  “Caring for individuals from diverse backgrounds is a daily reality for nurses and midwives, who are expected to provide care which is both clinically safe and culturally sensitive. (Williamson & Harrison 2010)” - **Pregnancy care for migrant and refugee women -** **Pregnancy Care Guidelines (Australian Government Department of Health 2020)**  “NSW Health understands the needs, experiences and identities of culturally and linguistically diverse communities in NSW” (p. 5) - **NSW Plan for Healthy Culturally and Linguistically Diverse Communities: 2019-2023 (NSW Health 2019)**  “...highlights specific approaches to pregnancy care for a range of groups, with a focus on improving the experience of antenatal care for Aboriginal and Torres Strait Islander women, migrant and refugee women and women with severe mental illness”. **Clinical Practice Guidelines: Pregnancy Care (Australian Government Department of Health 2020)**  “...focuses on providing women with family-centred, quality care during pregnancy, birth and the postnatal period”. NSW Health Maternity Care Policy **(New South Wales Ministry of Health 2020)**  “...involvement of multicultural health workers may be a consideration in the assessment of migrant and refugee women”.  “Consider language and cultural appropriateness of any tool used to assess psychosocial risk” **Clinical Practice Guidelines: Pregnancy Care (Australian Government Department of Health 2020)**  “You can reduce the risk for your baby by following these safe sleeping recommendations every time you place your baby to sleep:   - Place your baby on their back to sleep. - Your baby should have their own cot that meets the Australian safety standard and has a firm, well fitted mattress. - Sleep your baby in your bedroom at night for the first six to 12 months of life. - Do not let your baby sleep on the couch or an armchair, especially with another person. - Make sure your baby's head and face cannot become covered while sleeping to prevent suffocation or overheating. Tuck in sheets and blankets or use a safe infant sleeping bag. Do not use a doona, cot bumper, mattress padding, sheep skin or leave soft toys in the cot. - Dress your baby to be comfortably warm but not hot, to avoid overheating - Breastfeed your baby for the first six months where possible. - Don't smoke during pregnancy or after your child is born and don't allow anyone to smoke near your baby. - Make sure anyone who looks after your baby understands these safe sleeping recommendations”.   NSW Health Maternity Care Policy **(New South Wales Ministry of Health 2020)**  “Before using a baby carrier, sling or pouch, parents and carers should be aware that babies who are born premature, of low birth weight, are unwell, or are under four months of age are at greater risk of suffocation.” NSW Health Maternity Care Policy **(New South Wales Ministry of Health 2020)** | **Western approach can be inappropriate for clients:** “As professionals here in health, we worked from a Western perspective and from a Western lens, and it’s not necessarily the right approach, and not necessarily the right strategies or the right path to take” (Provider 21).  **Dilemmas around cultural practices that may increase the risk of SID, e.g., co-sleeping jewelleries, crosses** “And, things like sleeping with your baby, we find that certain cultures do it more than other cultures. As long as they understand the SIDS risk. I always explain the SIDS risk and the dangers and the red alerts when you shouldn’t do it” (Provider 01). “What I find another challenge is because of different cultural upbringings, an issue like the cot has been the hot issue because a lot of the times from certain cultural backgrounds, babies don’t sleep in cots…” (Provider 06) “...we have strict guidelines with SIDS… So, I think education on all those kinds of rituals and practices would be very beneficial. Also, I don't know about implementation of how they can practice culturally-safe practice in the unit.” (Provider 16) “Understanding the cultural practices, like, such as co-sleeping and the importance of family support as well” (Provider 20).  **Dilemmas around dominating and/or aggressive husbands:** “...where you can see that the woman is in a culturally arranged relationship, where she is quite powerless, and she is obliged, as part of the marriage relationship, to comply with the husbands wishes, and the husband is dominating and aggressive and there’s clearly a problem, but she considers it normal, and you know it's not normal” (Provider 17).  **Discomfort with male staff:** “...husband was just having a hard time, you know, allowing a male doctor to examine his wife because culturally that’s inappropriate to have another male examine a woman”. (Provider 06) “Partners not wanting to leave the room for any examinations, and partners not wanting males involved in care” (Provider 17).  **Fathers more comfortable speaking to another male:** “I've found some of the cultures where if it’s more male-orientated, where the father might approach myself in a different manner to a female” (Provider 16).  **Need for understanding other culture and being aware of different cultural practices: “**I think basically it’s respect of other people’s culture and their differences and it’s being on a level of power, it’s not looking down on another culture. It’s having a safe and culturally appropriate place to meet someone”. (Provider 03) “Understanding their culture and being aware of different cultural practices. Caring for patients and families in an ethical way.” (Provider 16). “So, I think for me it’s always been having an open mind, firstly, being totally open, and understanding that that woman comes from a completely different background to me” (Provider 25).  **Need to be “extra kind” and “just compassionate” “extra warmth”:** “even though a nurse or they're doctors, you think they're very hard and very not compassionate” (Provider 1). “Kindness is very important. They need people to be kind to them, and they expect people to perhaps not be” (Provider 17). “But just being aware that they have been through a lot more than we will probably ever see in our lives, and just – yeah. Being extra kind. And, just compassionate” (Provider 18).  **Need for trauma-informed approach:** “...the first and foremost catchphrase is trauma inform because a lot of the refugees we have are from about 80 to 90 percent war-affected countries. So, trauma inform is the first and foremost thing you need to be aware of, and then being culturally informed and culturally aware is the word.” (Provider 01). “Lots of these women have been through a lot of trauma. And there’s a lot of – they’ve seen a lot and been through a lot. And, just trying to be – making sure we’re just – like, we’re aware of that: (Provider 18).  **Need for bilingual liaison officers/ante-natal groups/mother’s groups:** “We don’t have liaison officers in the hospital from different cultures. But if there was a service or something that would be suitable for that family’s needs”. In [metro area] they have bilingual obstetric liaison officer, bilingual. We, in our area, we don’t have this. That’s a big gap. That will make life very, I don’t say easy. It access. They make our client access what available at the hospital, and make people understand them in the hospital”. (Provider 21). “It’s just linking them in with those services that are culturally appropriate as well. I know that we have some different mother’s groups or playgroups that are multicultural or that are targeted to a specific cultural group as well” (Provider 22).  **Screening processes not culturally responsive:** “They need to look at a more culturally informed screening process for different cultures. Not just refugee clients but any, sort of like, migrants that are, that may be in the system and not understood from that perspective” (Provider 19). “Edinburgh. Yeah, it’s just not culturally appropriate. No. It’s just translated into different languages” (Provider 21). “The Edinburgh Depression Scale. The EDS. Because if you ask a Muslim client, do you have thoughts of suicide or self-harm, they’re going to say no, because it’s against their religion to self-harm. So, they will not verbalise it, because if I do, then I’m talking about committing a sin” (Provider 24). |
| **Organizational Accountability/ Cultural Competence Support + Training**  “Health providers, services and health organisations are competent and are supported to become culturally  competent and to deliver high quality services” (pg. 27) - **NSW Refugee Health Plan 2011-2016**  “Our organisation’s leaders promote and improve cultural responsiveness  -Our organisation assesses the cultural responsiveness of our staff and addresses gaps. We embed cultural responsiveness into our wider training activities and our approach to providing services  -Our organisation provides services and has designated positions to work with and respond to the needs of priority culturally and linguistically diverse communities, including people from refugee backgrounds” (p. 5) - **NSW Plan for Healthy Culturally and Linguistically Diverse Communities:**  **2019-2023 (NSW Health 2019)**  “Advise parents that antenatal education programs are effective in providing information about pregnancy, childbirth and parenting but do not influence mode of birth” **Clinical Practice Guidelines: Pregnancy Care (Australian Government Department of Health 2020)** | **Compulsory online training on cultural competence** – very general and not helpful; “it’s just like a one-off thing”; “online isn’t sufficient” (Provider 5). “One of the compulsory trainings online is the cultural competency. It is about different cultures. However, I do find that a lot of the focus is not really on the different cultures, it’s more broad” (Provider 4). “When we all start our jobs, we have cultural competency trainings that we do on the computer, and I think it’s mostly online” (Provider 6). “We have what's called cultural competency training, but not in every culture. It's not good training. It lacks – what does it lack? It's perfunctory and if you understand this, you’ll be able to manage these situations” (Provider 17).  **Need for formal and informal education:** “educating staff members around respecting other people’s cultures. “The best training is asking the women about their culture and showing that genuine interest about what is appropriate for them and what is not and trying to incorporate that” (Provider 4). “I think it needs to happen possibly more, and to everybody as well. Not just to one area. I think the whole hospital needs to be educated and be more” (Provider 18). “I think that needs to be in a relatively intimate group, and it needs to be with women who come from cult backgrounds. And I would love to see that as a half-day study day. Hear some stories of women” (Provider 25).  **Lack of training to work with refugee and migrant women;** “I haven't done any training myself in refugee health. I think it’s a gap.” “I worked in mainstream areas where it’s almost really hard to get sent off to a course. You have to justify it” (Provider 1). “I feel that the nurses in this area, we don’t have a lot of knowledge and understanding of refugees and their background” (Provider 16).  **Understanding cultural practices around birthing:** “There is a lot of culture issues with birthing, especially after birthing. And expectations that you get a lot of support from your family, from the community. And many people, they don’t have family.” (Provider 21). “Understanding of different cultures. Also, different cultural practices with birthing, breastfeeding, cultural practices surrounding placenta usage as well” (Provider 16).  **Wish for more training (culture focus vs refugee-related issues focus):** “I think it’s quite meaningful to give nurses the education of the history, of how they became displaced” (Provider 16). “I don’t think there’s a particularly good understanding, at all, of refugees and asylum seekers, even on the basic level of what the difference is there” (Provider 2).  **Budget constraints limit the emphasis on cultural competence:** “budget is a constraint” (Provider 16). “That’s one thing that comes down to budget” (Provider 18). |
| **Cultural Competence of Mainstream Services**  “Many refugees are routinely referred to mainstream health services such as maternal and child health services (…) It is important that these and other mainstream services are culturally competent and understand the health needs and issues of refugees” (pg. 27) - **NSW Refugee Health Plan 2011-2016** | **Hospital helping to fulfil religious wishes:** “sometimes it’s helpful to know certain cultural practices like a Muslim woman prefers for their loved ones who have died to be buried on the same day” (Provider 6). “We had to use our tutorial room, what we call – we do it more for education and ward rounds, because there were – in that room, I think we had about 20 people and that was not even everybody. There was probably at least 30 people that were here, and so, I don’t think we always had space to accommodate” (Provider 5). “I don’t know if there's any area, you know, to pray for different faiths and cultures as well” (Provider 16).  **Systemic barriers and inherent limitations of hospitals to provide culturally competent service:** “I think that there are those systemic barriers to that always happening”. I think that hospitals have limitations” (Provider 5). |
| **Racism/Prejudice/Bias/Discrimination**  The policies do not address racism explicitly, but it comes up in four interviews | **Experience of racism/discrimination:** “I would feel that a lot of the times, there is that racism in the background” (Provider 4). “I try and just work with them and even if I feel really like this is straight racism...” (Provider 2). “There's a lot of racism in health sectors. I've seen it quite often working as a midwife. It’s quite distressing because I'm from a non-English speaking background and I can understand” (Provider 1).  **People on visas experience a lot of prejudice**: “There’s constant misunderstanding of what people’s visa status is and what that actually means. You look at it and think, they’re seeing someone, but really are only seeing the body in front of them. They’re not seeing the wider picture”. (Provider 02). “Prejudice. People who are here on a temporary visa, or who are resident aliens, or who are married to an Australian citizen, but they are not an Australian citizen” (Provider 17).  **Judgment and harmful way of talking in multidisciplinary teams:** “A lot of judgments around – parenting is such a space where people feel like they can pass judgement”. (Provider 05). “She’d just done the usual recommendations that were culturally inappropriate, to begin with. But also, didn’t acknowledge the fact they had no money” (Provider 2). “Needing support and skills and knowledge rather than judgement” (Provider 19). |

| **Improving Access and Quality of Services** | |
| --- | --- |
| **Policies say this:** | **Providers experience this (selected examples):** |
| “To provide high quality **specialised refugee health services**” (pg. 2) - **NSW Refugee Health Plan 2011-2016**  “NSW Health has strategies in place to improve **access and quality of care** for all people from culturally and linguistically diverse backgrounds, particularly people with vulnerabilities (Outcome 1):  - Our organisation routinely considers the health needs of culturally and linguistically diverse consumers, their carers and their families in the development and review of strategic plans, clinical and non-clinical service plans and relevant policies  -Our organisation has systems and processes in place to support assessment, care planning, care delivery and transfer of care that meet the cultural and linguistic needs of consumers” (p. 5) - **NSW Plan for Healthy Culturally and Linguistically Diverse Communities: 2019-2023 (NSW Health 2019)** | **Providers not aware of role of refugee specific services or multicultural health services:** “Structurally, within health, we don’t fit anywhere...If you don’t slot into the right place, you’re overlooked” (Provider 02). “We have services like Refugee Health, which is really great that we link families in as well. The barrier is that they’re not accessing the support that they need, that healthcare that they need.” (Provider 05). “Maybe we could utilise the Multicultural Support Services a lot more. I didn’t even think about that, ‘cause it’s not something that we use often” (Provider 25).  **Nurses have to do case management and take on a holistic approach:** “It’s probably a much broader role than the typical child and family health role. There’s a lot more of a welfare focus as well, a much more holistic focus” (Provider 2). “It’s fighting bureaucracy, talking to health professionals or admin people and finance departments” (Provider 3).  **Assisting to navigate the system:** “The challenges, I guess is just helping them to navigate the system that might be very foreign – well, it is foreign to them and so unknown” (Provider 5). “...we’re dealing with post-traumatic stress disorder plus we’re trying, our clients are trying to navigate life in a new country, find housing, work, Centrelink. You know. Healthcare, Medicare, all the, all the things that you navigate when you first move to a new country. And the healthcare system...” (Provider 24). |
| “To foster the provision of **high quality mainstream care** to refugees” (pg. 3) - **NSW Refugee Health Plan 2011-2016**  “NSW Health has strategies in place to improve **access and quality of care** for all people from culturally and linguistically diverse backgrounds, particularly people with vulnerabilities (Outcome 1):  - Our organisation routinely considers the health needs of culturally and linguistically diverse consumers, their carers and their families in the development and review of strategic plans, clinical and non-clinical service plans and relevant policies  -Our organisation has systems and processes in place to support assessment, care planning, care delivery and transfer of care that meet the cultural and linguistic needs of consumers” (p. 5) - **NSW Plan for Healthy Culturally and Linguistically Diverse Communities: 2019-2023 (NSW Health 2019)**  “At the first contact with a women during pregnancy, make arranged for the first antenatal visit, which requires a long appointment and should occur within the first 10 weeks” **Clinical Practice Guidelines: Pregnancy Care (Australian Government Department of Health 2020)**  “Early in pregnancy, provide women with information in an appropriate format about the likely number, timing and content of antenatal visits associated with difference options of care and the opportunity to discuss this schedule” **Clinical Practice Guidelines: Pregnancy Care (Australian Government Department of Health 2020)** | **Need for more women doctors:** “To have more women doctors, I mean also to train the male doctors, who are working in OB-GYN can be aware that, especially with working with them in [name of the location], that’s just some of the things that you might facing with and don’t take it personally, but that’s just the way culturally they operate” (Provider 6).  **Short-staffed:** “Our entire team is heading towards burnout. Not just individuals, but the entire team. We are struggling. We need more staff.” (Provider 4). “Although, they might be referred through our meetings for refugee migrant status and if there’s no other psychosocial vulnerabilities, if it’s just limited support network, then we may not become involved. Whereas, if we’re a fully staffed team, if we had more social workers, then we potentially could”. (Provider 5). “Lots of the time we’re on skeletal staff. And maternity is priority obviously when they’re being discharged, rather than the antenatal, which lots of the time I think this is when we should be seeing the woman. To see and support them through and try to get everything ready...” (Provider 18).  **Lack of coordination of services:** “sometimes a referral to other services as well, but that’s just been really difficult”. (Provider 6) “…lack of communication, and that lack of coordination of support services” (Provider 25). “Even getting cooperation from other areas in the health system to work with you with a family, I think, is probably for me, at the moment, one of the really big frustrations” (Provider 2).  **Providers working in silos, e.g., OB’s interested in reproductive care only;** “They just deal with obstetrics, so they don’t even think about mental health. They wouldn’t even think about screening a woman for domestic violence” (Provider 25).  **Inconsistent coordination of care and referral pathways:** “Even getting cooperation from other areas in the health system to work with you with a family, I think, is probably for me, at the moment, one of the really big frustrations” (Provider 2).  **Limited housing services available**: “A barrier that has come up multiple times, and we struggle with as professionals, is the housing and accommodation for refugee women or women with no visa, so asylum seekers as well. If they’re leaving a violent relationship, they have nowhere to go” (Provider 4).  **Social workers in hospitals advocating for women:** “With the baby goods, we’ve been trying to be creative in terms of trying to get services that will help them with food or bills” (Provider 6).  **Programs needed to address social isolation:** “they only do one home visit most of the time or one phone call, even”. (Provider 6) “...with the referrals that we get in our Safe Start meetings, I mean, 80 percent of them the referrals to social work, 80 percent of them are experiencing social isolation” (Provider 6).  **Women don’t understand the roles and responsibilities of the providers:** “I think in just understanding what we as health social workers do. Them understanding who we are, what we do” (Provider 18). “I try to explain what different organisations do and what we do and how we can help” (Provider 16).  **Delay accessing care due to lack of knowledge about service access:** “The other barrier is that it delays women coming in for antenatal care and for treatment and the broader impact of delaying that treatment” (Provider 5). “I find that they don’t come to antenatal appointments until late in their pregnancy because they don’t have the money to pay for antenatal care and they’re afraid, and they’re working so hard until they’re in their last trimester and usually in a retail job or a physically demanding job in a factory and it’s endangering their life and the baby’s life” (Provider 6).  **Women arrive late or miss medical appointments:** “...and then obviously, women get judged if you don’t. Why haven’t you booked in? Why haven’t you attended your appointments? You know, and it’s all these. And then, you know, it gets flagged.” (Provider 18). “Also, when mothers to be present late in hospitals, hospitals usually refer them to social worker and then to other support services” (Provider 20).  **Women don’t have transport to get to appointments** “Mums coming to a hospital, not being able to afford transport…Some women are here on their own and can’t drive”. (Provider 16). “Just a practical – “Who can transport you up here?” You know. There’s gaps in the system…do they have the means to even get to the hospital?” (Provider 18).  **Lack of baby-sitting services to assist women with making to the appointments:** “...their appointment might be at one o’clock – this is in general as well. And they won’t be seen by a doctor ‘til two or three o’clock. You know, like – and then, they may have a child to pick up at three o’clock. You – you – there’s so many different factors...” (Provider 18)  **Lack of orientation to health care services, e.g., prenatal services:** “The system is, is that all women who intend to birth here, have to book in here, but of course, there are women who fall outside of us, and never book in, or have no antenatal care. They tend to turn up by ambulance... They don’t speak the language, they can't understand, they don’t know how to sign forms...” (Provider 17)  From a service perspective, I think we can do so much better. I can do – I think we can do so much better for those women” |

| **Taking an Individualised Approach** | |
| --- | --- |
| **Policies say this:** | **Providers’ experience this (selected examples):** |
| -“taking the time to establish rapport and trust with each woman”  -“consulting the woman about whom she would like to involve in her care”  -“if necessary, advocating on her behalf so that she receives appropriate care throughout pregnancy”  -“respectfully exploring cultural and personal understanding and experience of pregnancy and appropriate self-care in pregnancy, and encouraging the woman to discuss anything she is worried or unsure about” -**Pregnancy care for migrant and refugee women -** **Pregnancy Care Guidelines (Australian Government Department of Health 2020)**  “Determine the schedule of antenatal visits based on the individual woman’s needs” **Clinical Practice Guidelines: Pregnancy Care (Australian Government Department of Health 2020)** | **Taking time to establish rapport:** “so that they can understand and also build their trust and orientate them to what the service is about” (Provider 1). “It’s really about establishing trust and me introducing myself and just getting anything that’s on their mind addressed…” (Provider 19).  **Often limited intervention/short-staffed impacts on building rapport women**: “I only have 30 minutes with that woman. What am I going to do in 30 minutes when this is so complex?” (Provider 25). “Where you can build rapport, you’ve got better outcomes for mothers and babies. Where it's short intervention, that’s really, really difficult to get right” (Provider 14).  **Restrictions in providing continuing care:** “We’re not even able to offer that ongoing therapeutic intervention postnatally to women unless they’re under the significant domestic violence model of care” (Provider 4). “I mean I worked in mainstream and it’s just totally different to working here. Working here you feel the vulnerability a lot more intense and more acutely” (Provider 01).  **Mandatory reporting sometimes in conflict with cultural practices:** “...even getting the midwives with the interpreter about co-sleeping and the dangers of it. You know, SIDS, and just the education about it. And, you know – and, also if she didn’t understand that as well there’s child protection. We can’t allow her to go home to this. Like, we need to be reporting it then, if she chooses to take this baby home to this where there is no bedding for the baby” (Provider 18). “...first of all, trying to understand the women’s culture and what that would be like. Keeping in mind that we have professional obligations. We are mandatory reporters, and we have duty of care” (Provider 25) “we are mandatory reporters, so that is when we face that ethical dilemma. We know that this is a normal and safe practice in their culture, but it is not a safe practice in our culture” (Provider 4).  **Organisational processes and procedures vs women-centred care:** “midwifery care is provided from a woman’s centred perspective”. However, the logistics of providing care to women of other cultural groups, is often restricted by those things like politics...” (Provider 17). “I love working with women and making those minor changes and minor differences in their lives. However, when I’m restricted in that, I feel quite powerless. I feel a tiny bit of what they’re feeling, in a way”. (Provider 4) |
| “Health organisations are responsive to people’s individual needs, language and culture” (Outcome 3) - **NSW Plan for Healthy Culturally and Linguistically Diverse Communities:**  **2019-2023 (NSW Health 2019)**  “Include psychological preparation for parenthood as part of antenatal care as this has a positive effect on women’s mental health postnatally” **Clinical Practice Guidelines: Pregnancy Care (Australian Government Department of Health 2020)** | **Responding to complex/multiple needs is challenging.** **More support and supervision needed when dealing with complex cases:** “I felt I had no support.” (Provider 25) “So we are under a lot of pressure. We are very restricted. Thirty-five clients on one caseload is a lot. Keeping in mind that they’re all complex” (Provider 4).  **High risk only marked when there is medical risk (e.g., gestational diabetes), not mental health e.g., trauma**: I think as an organisation, as great as it is and I really do respect them in lots of ways, I was really disappointed that they didn’t acknowledge that complexity and then that impacts on our team’s ability to handle just the health issues. (Provider 3)  **Lack of trauma-informed practice:** ‘So, I said to my boss at some stage, “This is really hard.” And she said to me, “You’re just a midwife [name of the provider]. You just do your midwife care.” But I don’t think – that’s not my philosophy, that’s not where I come from.” (Provider 25).  **Important to acknowledge women’s resilience and remember the women are the experts on their lives:** “I think the women that we work with are even more resilient, and they teach us how to be resilient a lot of the time. It’s important for us to take a few steps back and just observe and learn from them as well, and not just be the expert because we are not the experts’ (Provider 04) “They have a plethora of experience and knowledge and skills that can apply to their new life here. And with a bit of support, they can achieve that” (Provider 19).  **Important to listen and show care: “**You need to find out what they feel are the problems, and how they think you could help, and take it from there” (Provider 2). “They just want to be listened to and acknowledge that things are difficult, and to be encouraged that they are doing a good job” (Provider 6). |
| “NSW Health supports people from culturally and linguistically diverse backgrounds to build their health literacy so they can be actively involved in decisions about their health (Outcome 2) -**NSW Plan for Healthy Culturally and Linguistically Diverse Communities:** **2019-2023 (NSW Health 2019)**  “Assisting parents to find an antenatal education program that is suitable to their learning style, language and literacy level may improve uptake of information” **Clinical Practice Guidelines: Pregnancy Care (Australian Government Department of Health 2020)** | Did not emerge in the providers’ interviews |

| **Using Interpreters** | |
| --- | --- |
| **Policies say this:** | **Providers experience this (selected examples):** |
| “The Commonwealth Translating and Interpreting Service (TIS) provides 24 hour a day, 7 day per week access to  telephone interpreting services through the Doctor’s Priority Line and on-site access to interpreters during the  day. Health providers working in public healthcare services may access a healthcare interpreter service at any time. Pharmacists may access the free TIS service.” (pg. 27) **- NSW Refugee Health Plan 2011-2016** | **Access to interpreters vital and TIS provides 24/7 access but issues with this service:** “for some languages it can be, are really different to find, some African dialects or the Burmese” (Provider 23). “Sometimes the lines are bad, the patient can’t hear very well on the phone” (Provider 24). “The process is not fully explained to the patient. It’s done in a rushed, like a tick box” (Provider 20). “If it’s an emergency it’s usually a brief conversation to tell the mother what is happening. Sometimes she still might not understand” (Provider 16). |
| “Our organisation seeks to ensure that consumers, their carers and their families can access professional healthcare interpreters when required and that infrastructure is in place to support efficient provision of services” (p. 5) - **NSW Plan for Healthy Culturally and Linguistically Diverse Communities:**  **2019-2023 (NSW Health 2019)**  “The care needs of migrant and refugee women can be complex. The first point of contact (e.g. first antenatal visit) is important and should be undertaken with an accredited health interpreter” **Clinical Practice Guidelines: Pregnancy Care (Australian Government Department of Health 2020)** | **Access to interpreters’ dependent on service provider:** “We will always have an interpreter, no matter what. No matter how late-minute appointments are booked in or how desperate I get I will always have an interpreter whereas mainstream this would never happen” (Provider 1).  **Access to face-to-face interpreters and limited appointment time:** “getting an interpreter is difficult and making sure we have them for the right amount of time – enough time” (Provider 18). “Better access to interpreters and to be able to provide an intervention quicker and not having to delay, that would be ideal” (Provider 4). “Sometimes the interpreters just stay for an hour”. “You can’t just condense the most important stuff into that time” (Provider 25).  **Encourage midwives/doctors to use interpreters to explain processes and laws e.g. co-sleeping: .** “I suppose within services it’s trying to get people to use interpreters, especially in private practice like specialists, they’re the worst even though they can get a free interpreter service” (Provider 03). “You know, just, at that time they can just see two or three person instead of, you know, just calling interpreter takes time for them and translation is time consuming” (Provider 19).  **Scheduling issues if interpreter is booked and woman is late for the appointment:** “they chronically come late, and the interpreter waits 20 minutes and leaves. That’s a common problem” (Provider 17). “You can imagine rescheduling, it's really difficult. In those circumstances, we may just bring the woman in, bring as many in as she wants. If we have to limit it, we ask her to limit it to someone who speaks English” (Provider 25).  **Interpreters provide cultural context:** “I look to the interpreters to give me feedback as to how I could relate to a client” (Provider 24). “The interpreters can be very useful because they can help you, culturally, to understand different situations when you have a good interpreter” (Provider 2).  **Providers not sure interpreters are conveying all information: “**I’ve had times where the interpreter and the patient are speaking to each other, and then I’m like, “Can you explain. I don’t understand” (Provider 18). |
| “Health professionals should take the initiative in organising for an accredited health interpreter wherever necessary, and reassure the woman of the benefits if she is reluctant” (p. 6) - **Pregnancy care for migrant and refugee women -** **Pregnancy Care Guidelines (Australian Government Department of Health 2020)** | **Women reluctant to use interpreter:** “Some of them say, “No, no, no, no, I don’t need an interpreter, it's fin,” (Provider 17). “I said, “Just give me a moment. I’ll call the interpreting service.” They just went, “Don’t need it” (Provider 25).  **Providers use Google translate to work around above issues:** “So, that’s quite a challenge to explain, without having an interpreter present, that we have to admit. So, I remember we used the phone, Google Translate” (Provider 25).  **Importance of using the same interpreter:** “I tried to just request for the same one, and I was seeing her a little bit more, like in an emotional support setting. So, it was really important to have an interpreter who can be consistent” (Provider 6). |
| “Do not use the woman’s partner, friends or relatives to act as interpreters unless absolutely necessary.” **- Pregnancy care for migrant and refugee women -** **Pregnancy Care Guidelines (Australian Government Department of Health 2020)** | **Family members may not understand medical terms and not translate correctly:** “when you have husbands translating that might not get you to the gist of what you need, so it’s really not from woman’s mouth” (Provider 25). “In some of the cultures, you can’t tell a person what’s really wrong. Right, a family member will not interpret correctly to the person what’s going on” (Provider 24).  **Only use family members if not discussing ‘stuff that is super important’ e.g., changing nappies:** “when it comes to stuff that’s not super important, then I can kind of gauge” (Provider 25). |
| “Suggest the use of an interpreter and, if the woman wants an interpreter, provide a female interpreter where possible” **- Pregnancy care for migrant and refugee women -** **Pregnancy Care Guidelines (Australian Government Department of Health 2020)** | **Discomfort with male interpreters:** “I had a male interpreter on the phone, aside from the fact that I actually punched in that I wanted a female interpreter” (Provider 25). “Sometimes the only translators available are male interpreters and it’s really awkward in a maternity setting to have a male interpreter” (Provider 6).  **Female interpreters may also provide emotional support to women:** “If you have the interpreter, because many, many clients tell me that interpreter spend many times in labour with the women they had the babies” (Provider 21). |
| “Consider confidentiality (e.g., in small communities, the woman may know the interpreter)” **- Pregnancy care for migrant and refugee women -** **Pregnancy Care Guidelines (Australian Government Department of Health 2020)** | Did not emerge in the providers’ interviews |
